# Supplementary material for: Atomic-level handedness determination of chiral crystals using aberration-corrected scanning transmission electron microscopy
Source: Nat Commun. 2020 Mar 27;11:1588. doi: 10.1038/s41467-020-15388-5 (PMC7101389; doi:10.1038/s41467-020-15388-5)
Supplement: Supplementary file 1 — Supplementary Information [file 41467_2020_15388_MOESM1_ESM.pdf]

Supplementary Information for

Atomic-level Handedness Determination of Chiral Crystals Using Aberration-corrected Scanning Transmission Electron Microscopy

Dong et al.

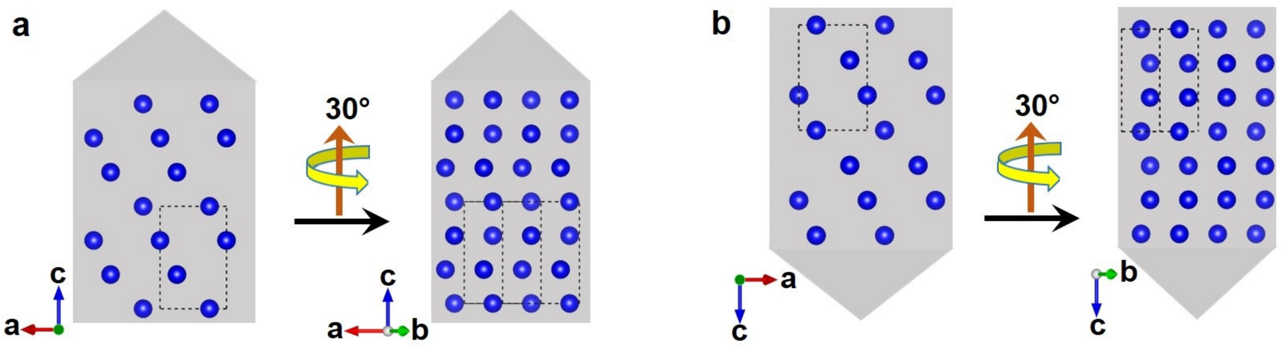

Supplementary Fig. 1 | Te structure projections with reverse *c* axis direction in a tilt series. (a) A tilt-series from [010] to [120] with the *c* axis up and (b) a tilt-series from [010] to [-110] with the *c* axis down. Blue spheres represent Te atoms.

As shown in the Supplementary Fig. 1, the bending direction of atoms along *c* axis is independent of the *c* axis direction.

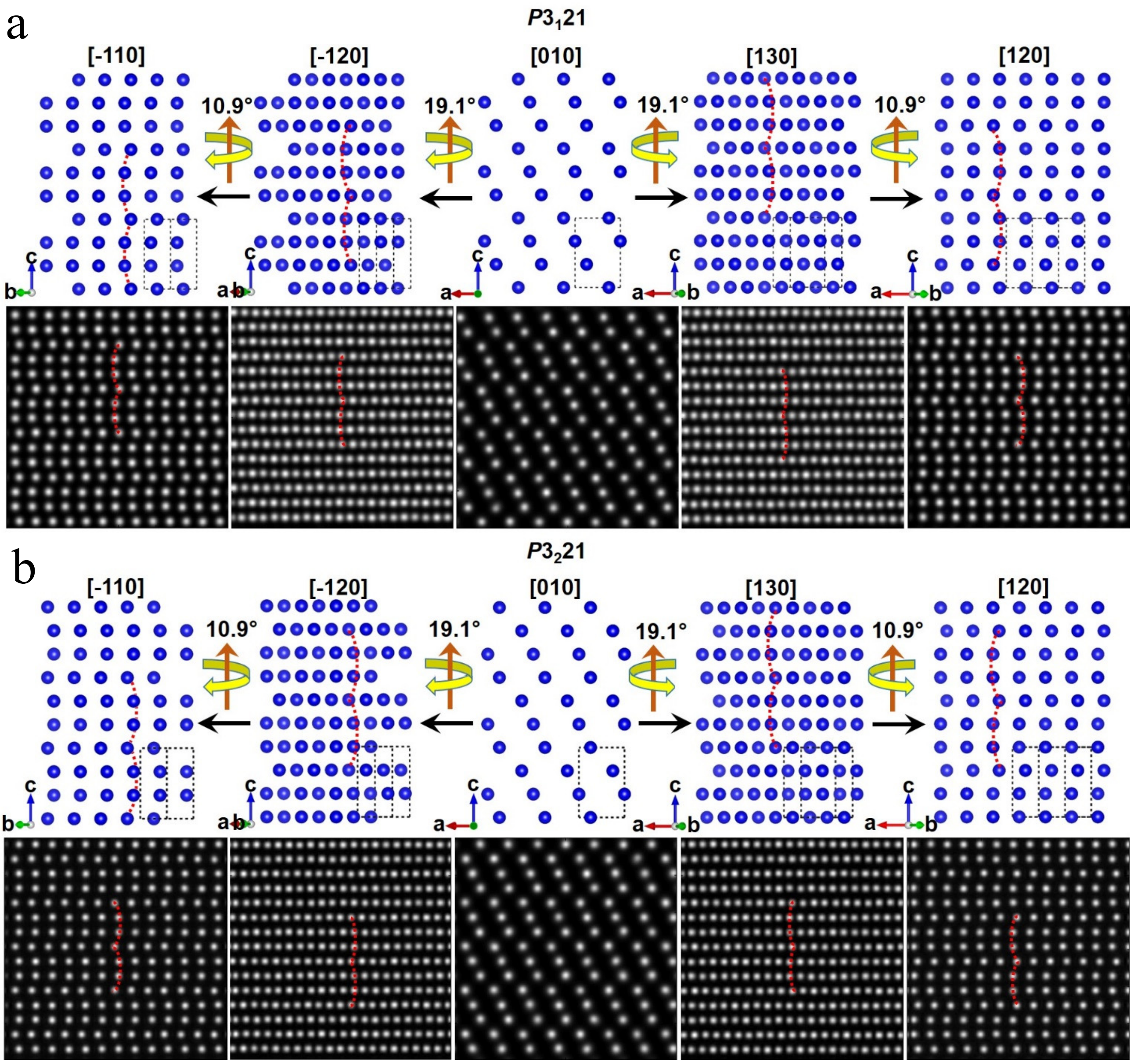

Supplementary Fig. 2 | STEM-ADF simulations of Te crystal structure along different directions in a tilt-series. The tilt-series of Te with (a) $P3_121$ and (b) $P3_221$ space groups, respectively. Blue spheres represent Te atoms.

Projections of Te crystal structure in a tilt series show difference for two enantiomorphic space groups. The difference can be observed in simulated STEM images, which proves the feasibility of this method in theory. Of note, at least two images were required for the correct determination of handedness.

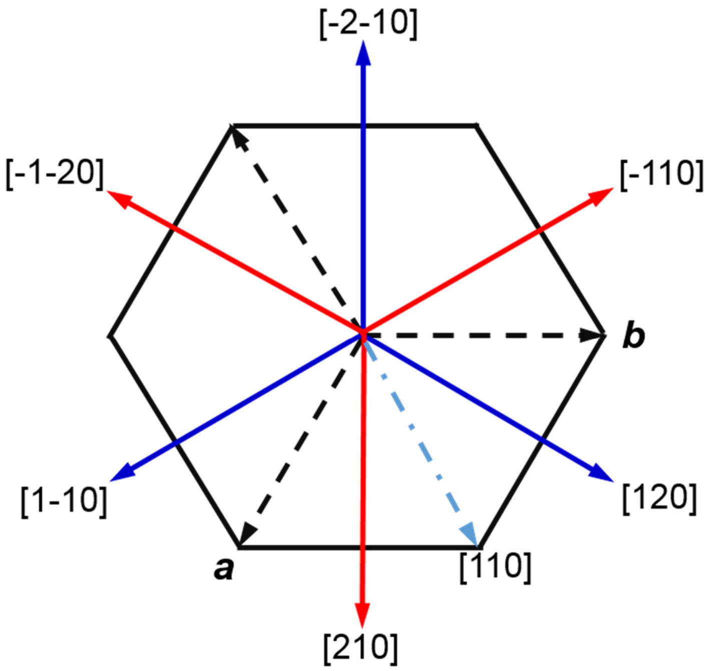

Supplementary Fig. 3 | The main zone axes perpendicular to the *c* axis.

For a trigonal crystal system, there are several zone axes can be chosen. The Supplementary Fig. 3 shows the symmetrically related zone axes perpendicular to the *c* axis.

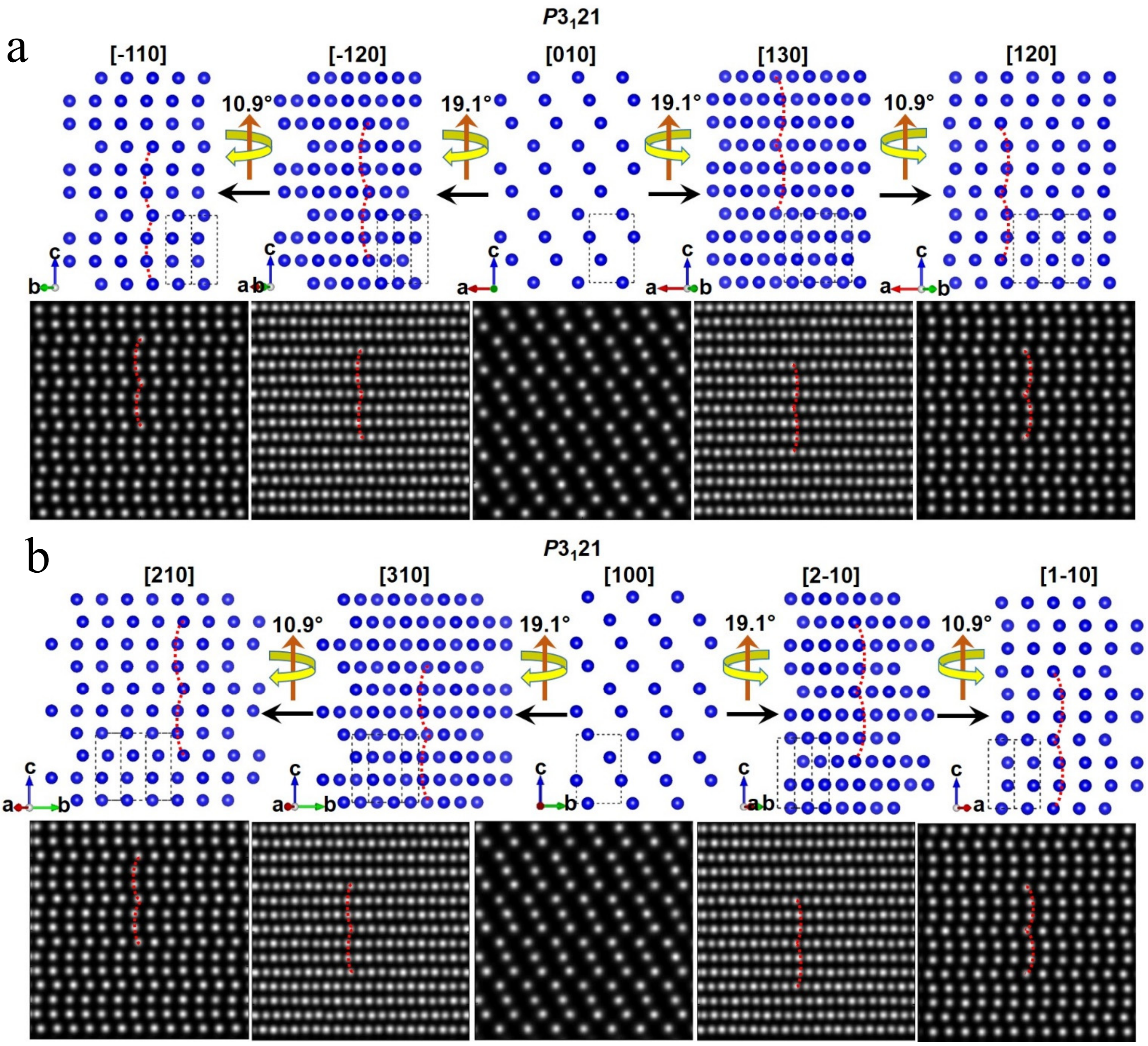

Supplementary Fig. 4 | STEM-ADF simulations of right-handed Te crystal structure along different directions in two tilt-series. A series of structure models and simulated images tilting from (a) [010] and (b) [100], respectively. Blue spheres represent Te atoms.

In trigonal system, [100] and [010] axes are symmetry equivalent. The two corresponding tilt series lead to same results.

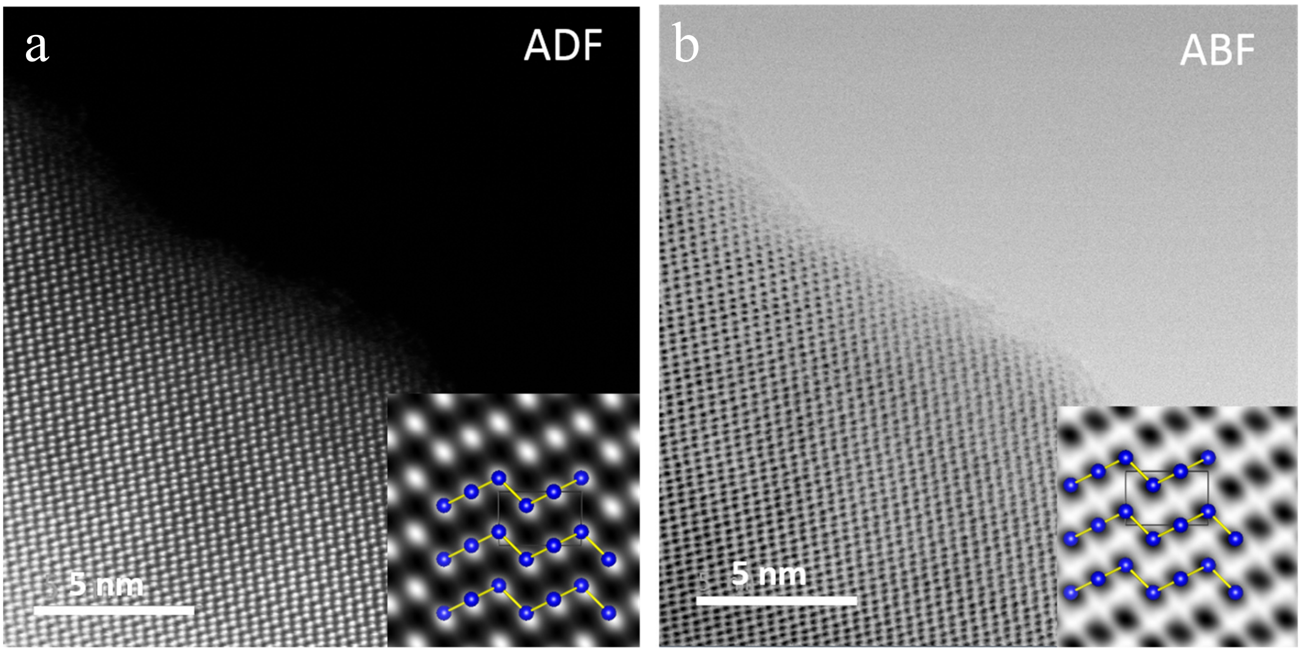

Supplementary Fig. 5 | STEM-ADF and ABF images of one Te crystal. (a) STEM-ADF and (b) ABF images were taken from the same crystal. Inset are simulated images; blue spheres represent Te atoms.

ADF and ABF images with atomic resolution can be recorded simultaneously. Both of them matches well with the simulated images and structure model.

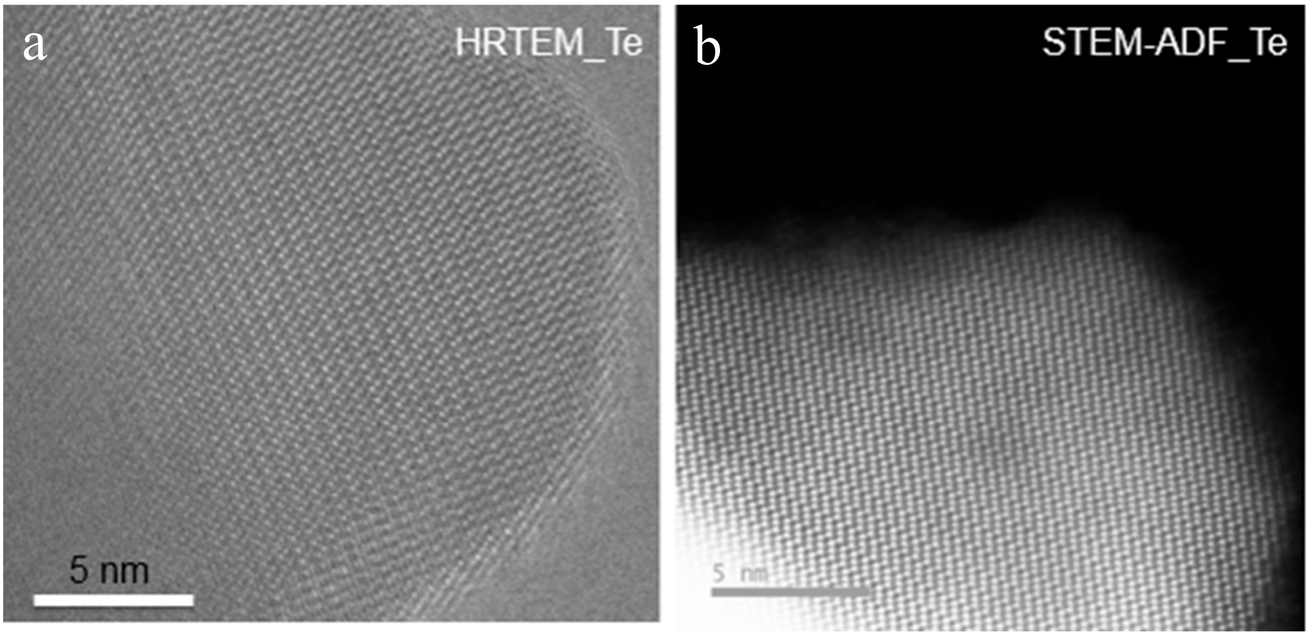

Supplementary Fig. 6 | HRTEM image and STEM-ADF image of Te crystal. (a) HRTEM image and (b) STEM-ADF image were taken from Te.

The two images were recorded using a GrandARM300F instrument with image and probe correctors at 300 kV. The contrast in HRTEM image is difficult to interpret due to the effect of contrast transfer function and crystal thickness. The contrast inverse can be observed even in the thin area of crystal. The STEM image taken from the same area shows much clear contrast and can be directly compared with the structure model. The comparison clearly shows that STEM shows advantages at atomic-level imaging.

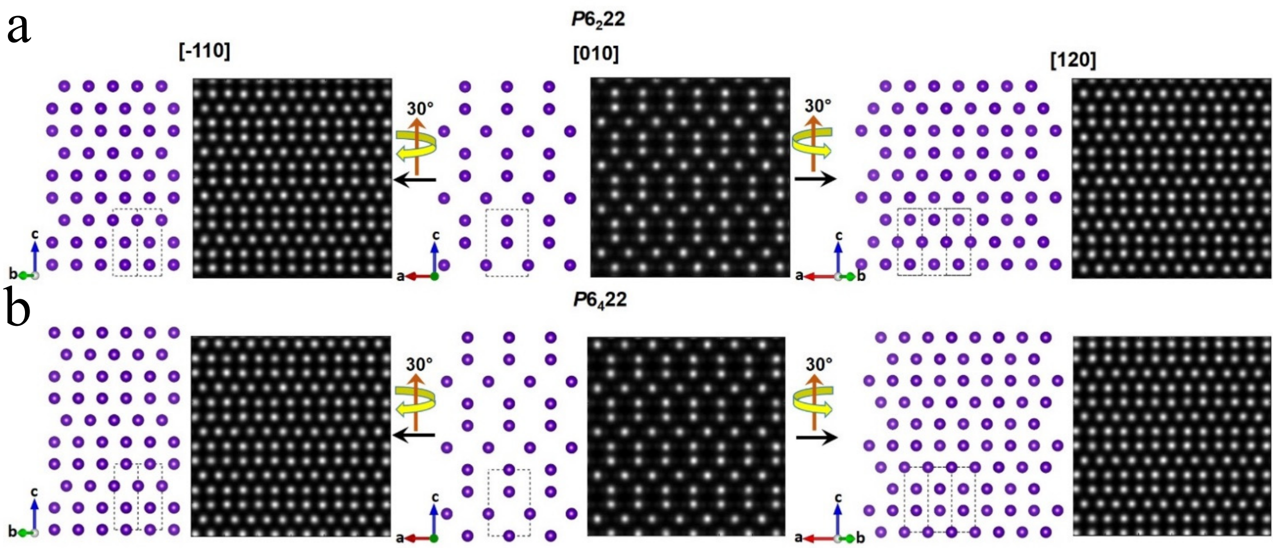

Supplementary Fig. 7 | STEM-ADF simulations of $TaSi_2$ crystal structure along different directions. The tilt-series of $TaSi_2$ with (a) $P6_222$ and (b) $P6_422$ space groups, respectively. Purple spheres represent Ta atoms; Si atoms are omitted in structure models for clarity.

There no difference between the two chiral structure projections after a 30° tilting from [010] axis as there is a six-fold screw along *c* axis.

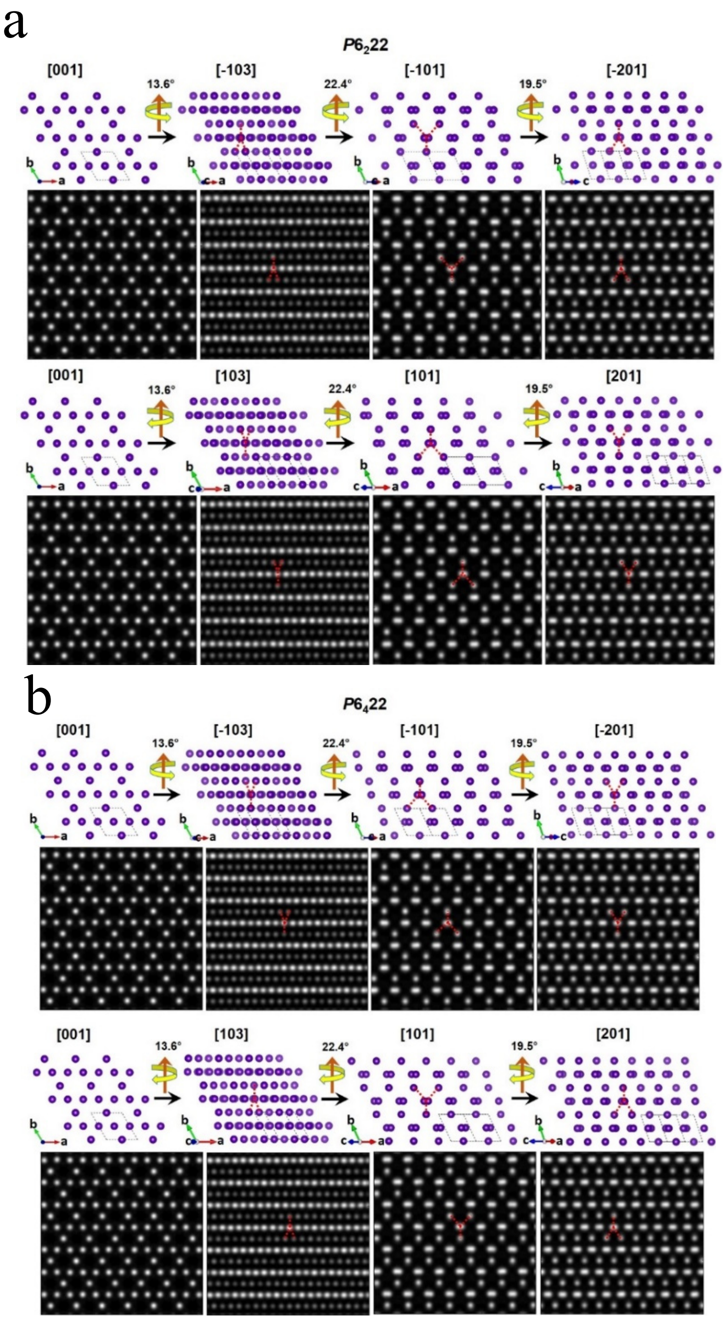

Supplementary Fig. 8 | STEM-ADF simulations of $TaSi_2$ crystal structure along different directions in a tilt-series. The tilt-series along different zone axes of $TaSi_2$ with (a) $P6_222$ and (b) $P6_422$ space groups, respectively. Purple spheres represent Ta atoms; Si atoms are omitted in structure models for clarity.

Projections of $TaSi_2$ crystal structure and corresponding simulated images in a tilt-series show the handedness can be determined by tilting the crystal around [120].

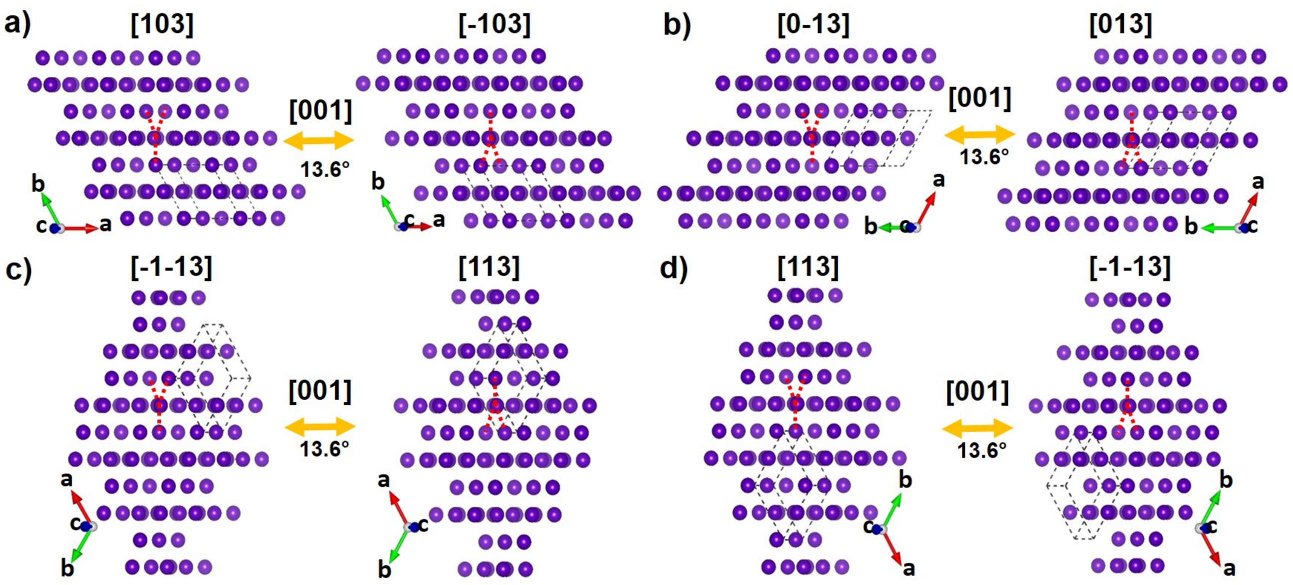

Supplementary Fig. 9 | Tilting of $TaSi_2$ structure to [-103] and [103] symmetrically equivalent directions. The tilting series are from (a) [103] to [-103], (b) [0-13] to [013], (c) [-1-13] to [113] and (d) [113] to [-1-13], respectively. Purple spheres represent Ta atoms; Si atoms are omitted in structure models for clarity.

$TaSi_2$ has a space group of $P6_222$ or $P6_422$. Therefore, [103], [0-13], [-1-13] and [113] are symmetrically equivalent. The tilting from [001] zone axis to one of the symmetrically equivalent directions will give the same structure projection.

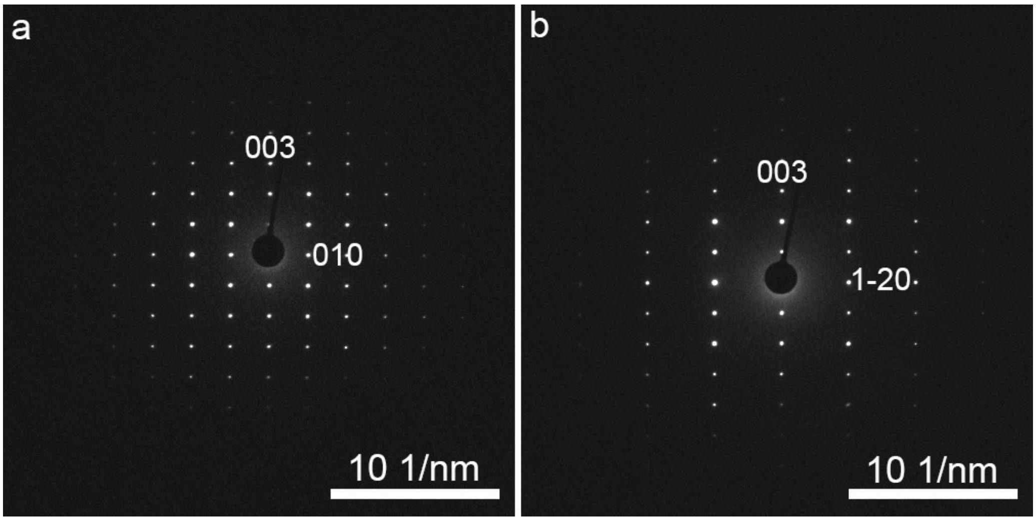

Supplementary Fig. 10 | Selected area electron diffraction (SAED) patterns of quartz. SAED patterns taken along (a) [100] and (b) [210] zone axes from a single crystal in a tilt series.

The determination of zone axes is based on indexing of SAED patterns. The observation of 001 and 002 reflections is because the crystal is thick and we used the whole crystal for SAED.

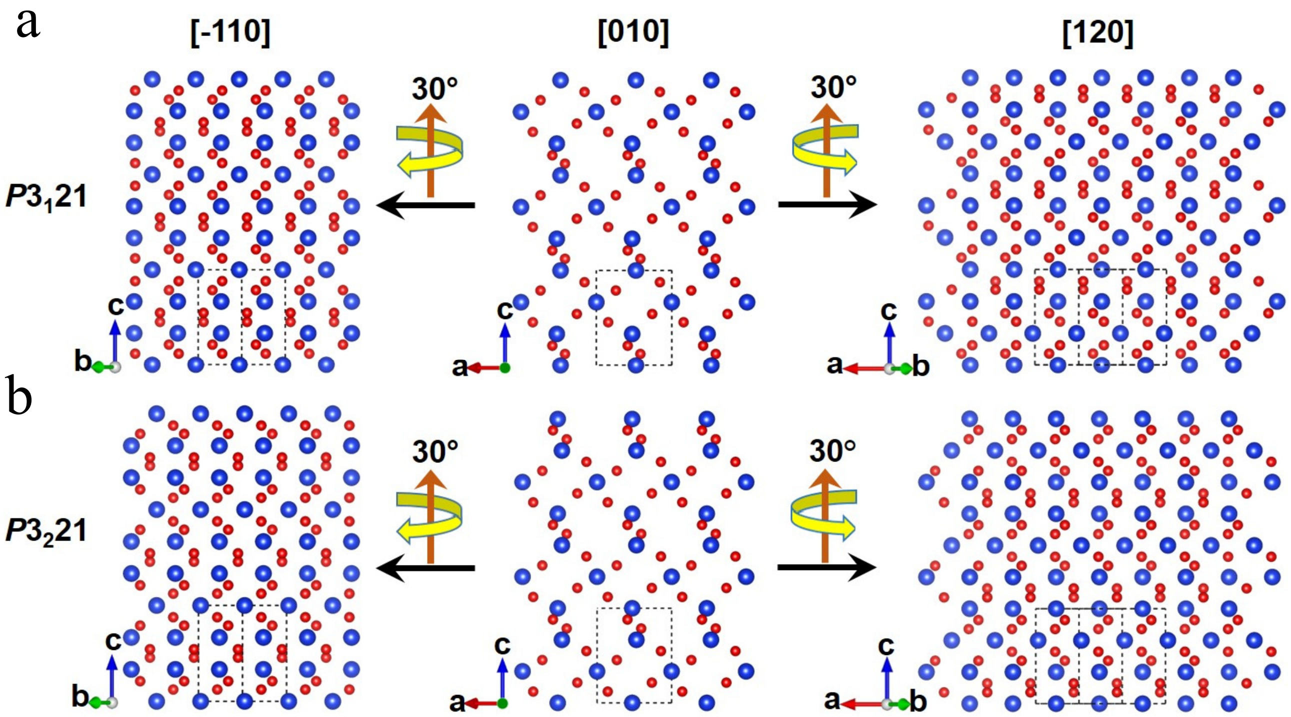

Supplementary Fig. 11 | Quartz crystal structure projections along different directions in tilt-series. Structure projections along different zone axes of quartz with (a) $P3_121$ and (b) $P3_221$ space groups, respectively. Blue and red spheres represent Si and O atoms, respectively.

Projection of quartz crystal structure in a tilt-series also show differences in two enantiomorphic structures.

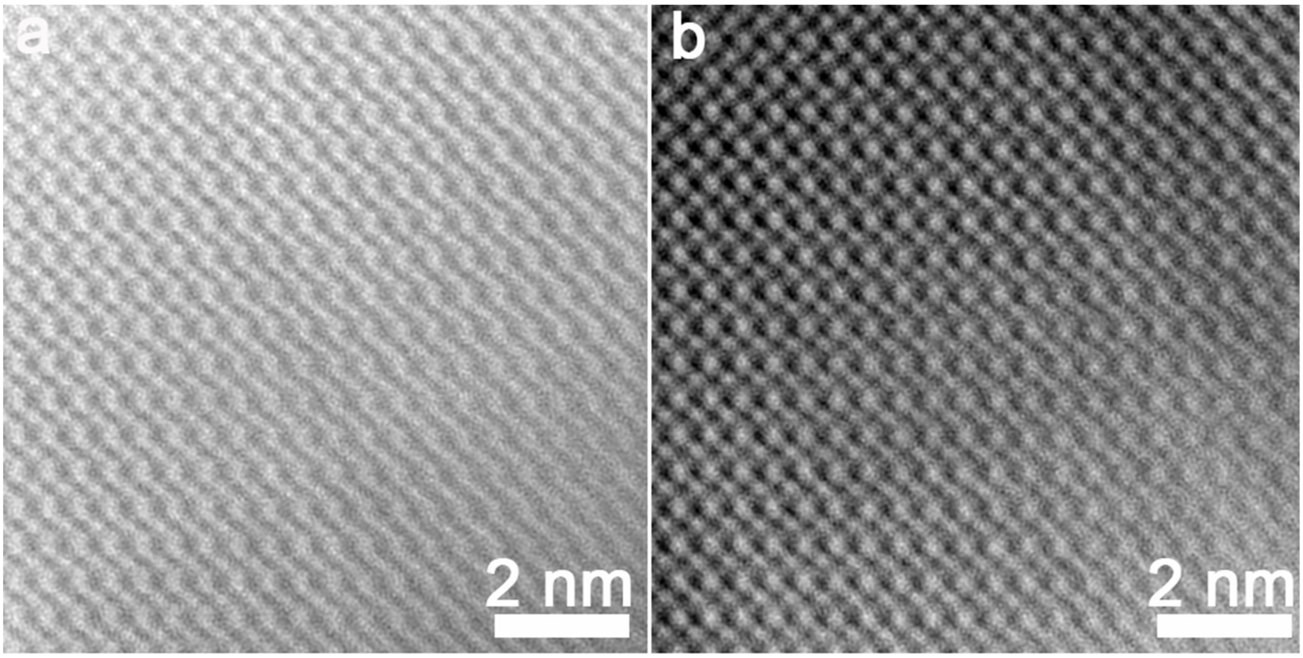

Supplementary Fig. 12 | STEM-ADF and ABF images of a quartz crystal along [010] direction. (a) STEM-ADF and (b) ABF images were taken from the same crystal.

Quartz is very sensitive to electron beams. High-resolution images can be obtained while the resolution can only reach 1.5 Å. It is difficult to directly compare the structure model with images.

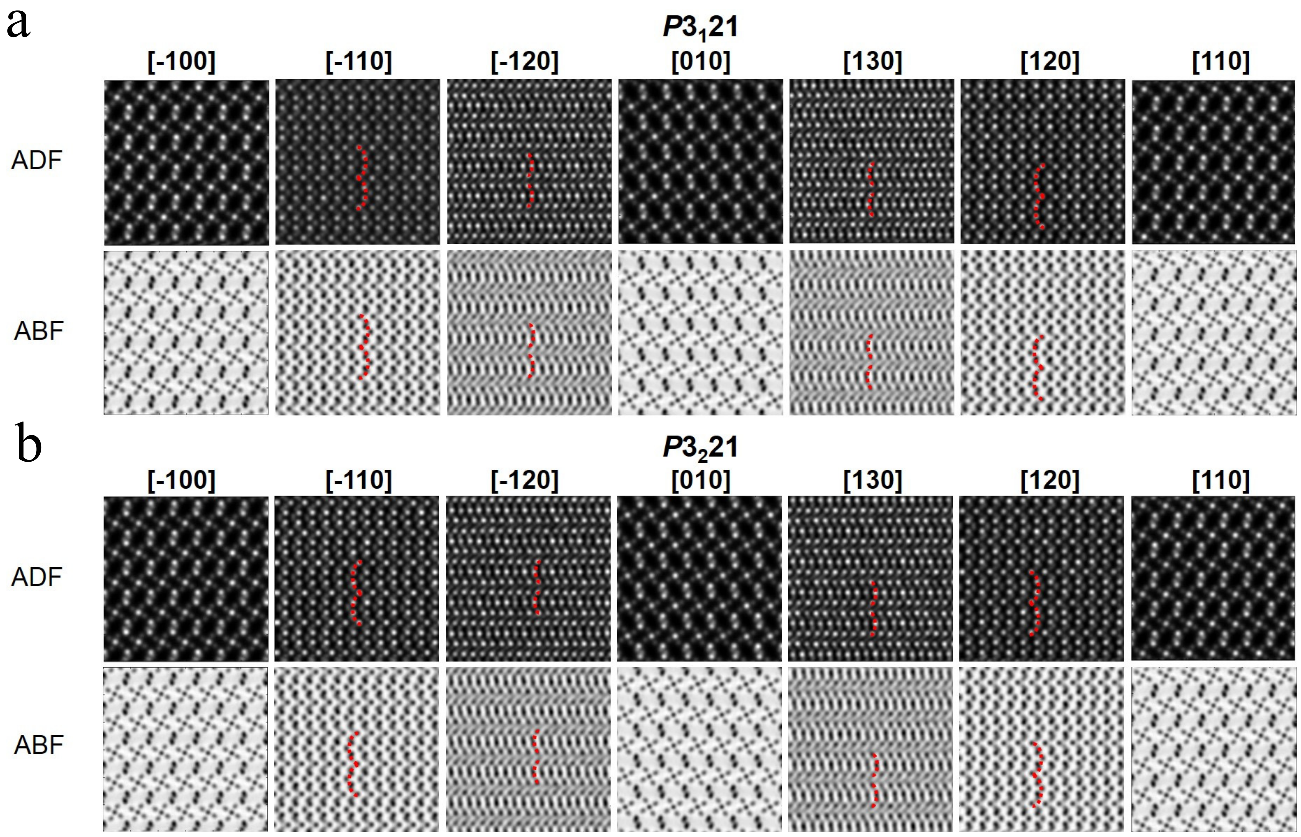

Supplementary Fig. 13 | STEM-ADF and ABF simulations of quartz crystal structure along different directions in tilt-series. Simulated images of quartz crystals with (a) $P3_121$ and (b) $P3_221$ space groups, respectively.

Both ADF and ABF images were simulated for quartz crystal. The contrast of light elements is clearer in ABF image than in ADF image.

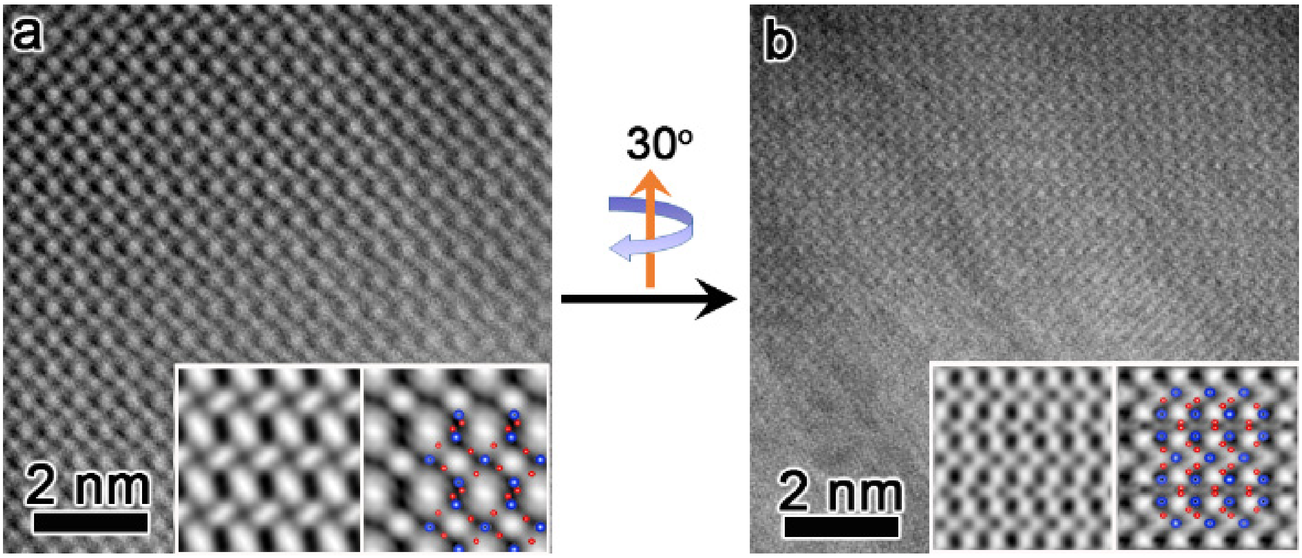

Supplementary Fig. 14 | A tilt experiment for a quartz crystal. STEM-ABF images of a quartz crystal along (a) [110] and (b) [120] zone axes (insets are simulated and symmetry-averaged images; blue and red spheres represent Si and O atoms respectively).

The resolution of STEM images of quartz is limited (~1.5 Å). The signal-to-noise ration is quite low in two images as quite low-dose conditions have been used (~0.9 pA). However, the crystal was still damaged quickly. Scanning distortion exists in supplementary Fig. 14b. The plane group symmetry *p*2 was applied to image along [110] and *pm* to image along [120] to increase the signal-to-noise ratio. The plane groups can be determined from the space group $P3_121$ or $P3_221$.

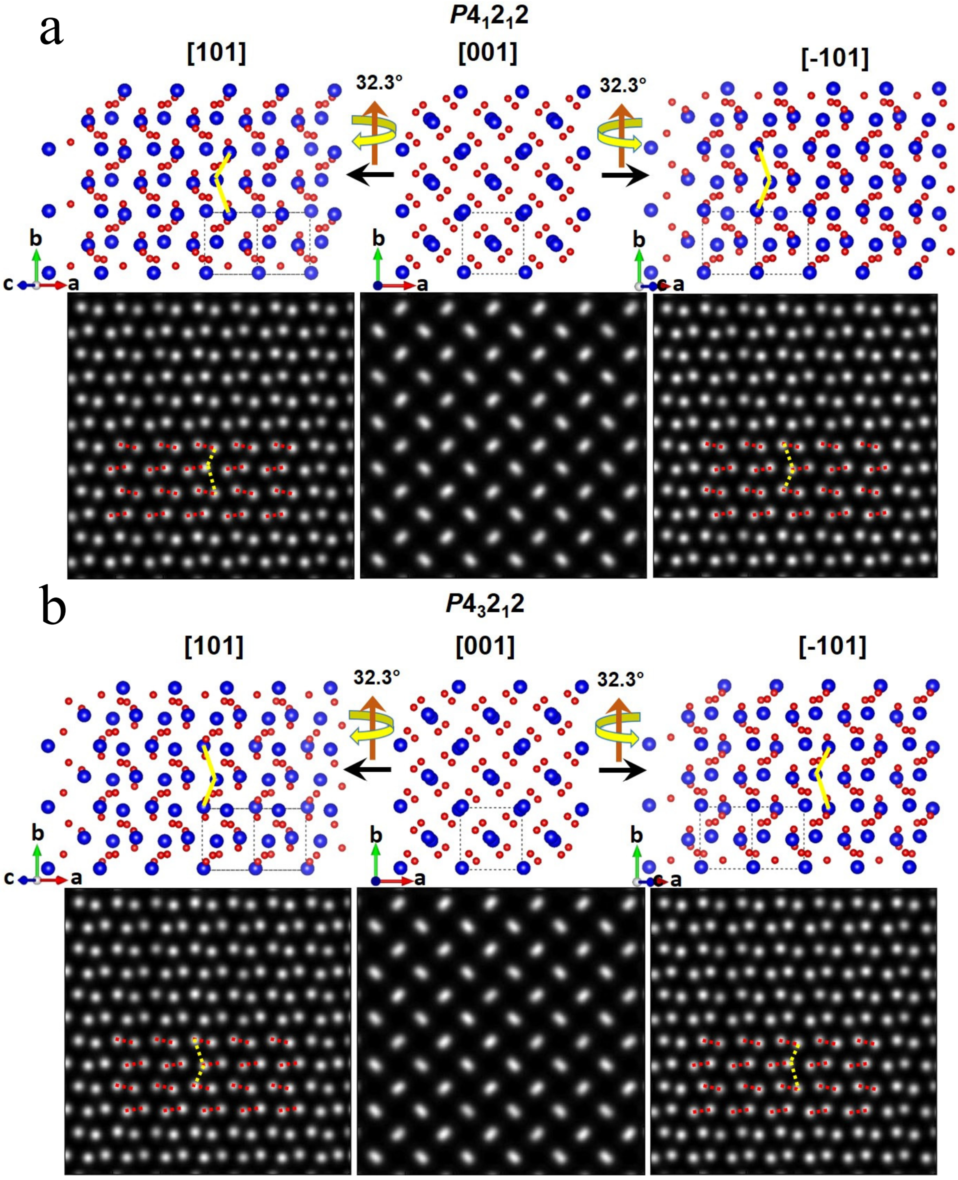

Supplementary Fig. 15 | Simulations of ADF images of TeO_2 crystal structure along different directions in a tilt-series. A tilt-series of TeO_2 structures with (a) $P4_12_12$ and (b) $P4_32_12$ space groups, and correspondingly simulated ADF images. Blue spheres represent Te atoms; red spheres represent O atoms;

Projections of structural models and corresponding simulated images of TeO_2 (space group $P4_12_12$ / $P4_32_12$) in a tilt-series show that the method can also be applied to crystals with tetragonal crystal system.

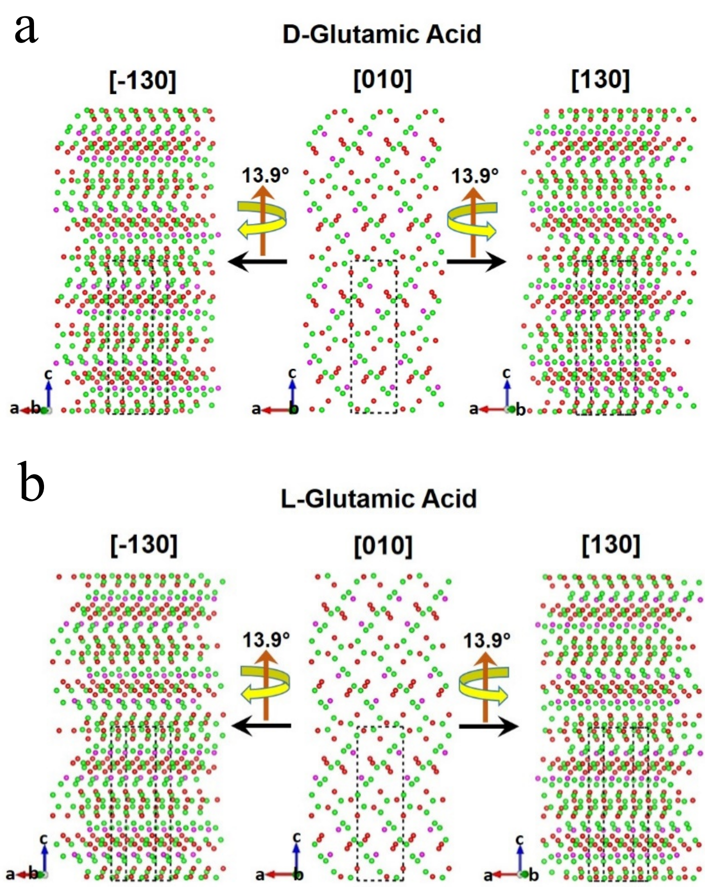

Supplementary Fig. 16 | Projections of *β*-glutamic acid crystal structures along different directions in a tilt-series. A tilt-series of structures is shown for (a) D-glutamic acid and (b) L-glutamic acid.

Projections of structural models of *β*-glutamic acid (space group $P2_12_12_1$) in a tilt-series show that the method can also be applied to crystals with orthorhombic crystal system.

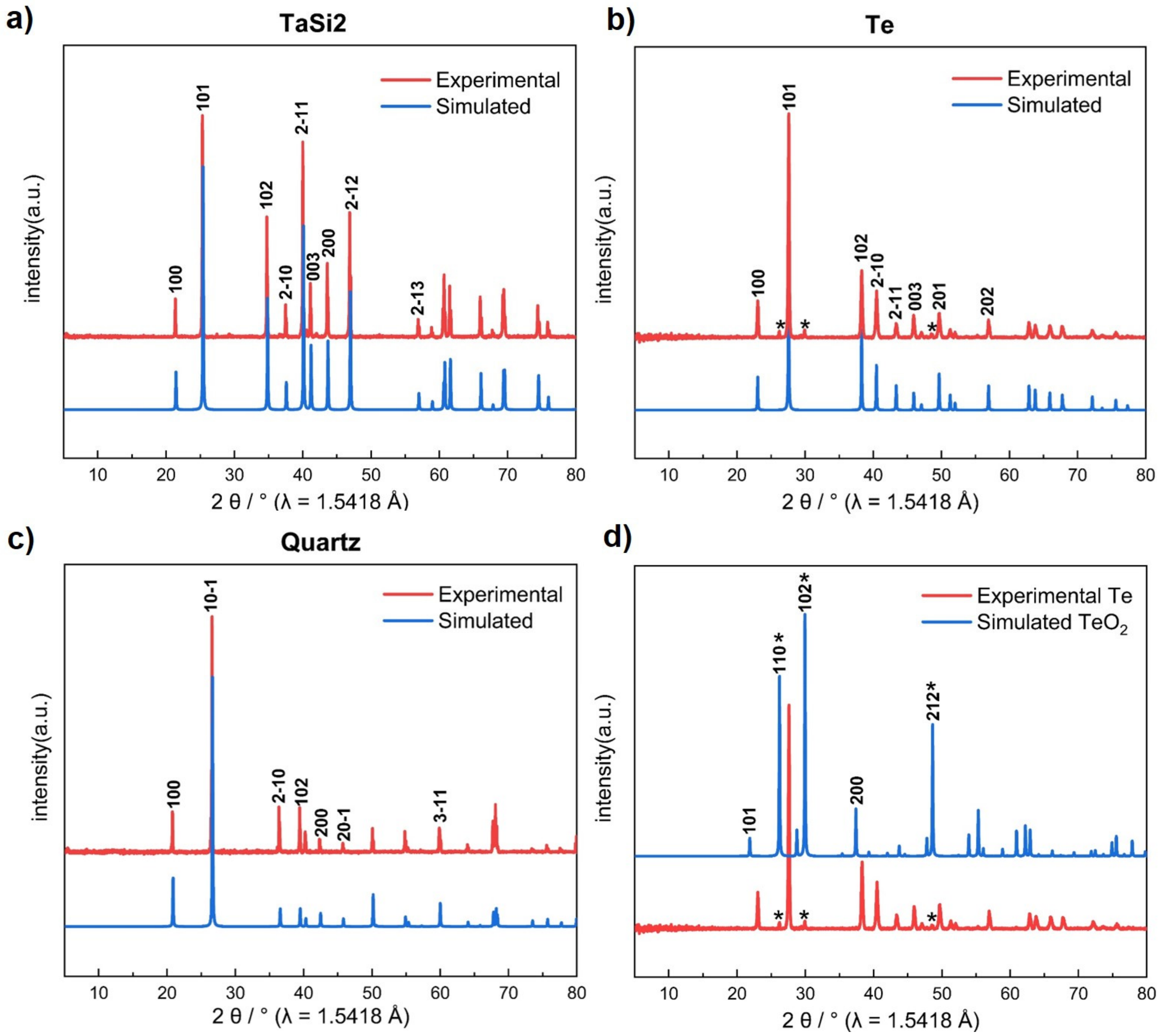

Supplementary Fig. 17 | Powder X-ray Diffraction (PXRD) patterns of TaSi2, Te, and quartz samples. Experimental and simulated PXRD patterns of (a) TaSi2, (b) Te and (c) quartz. (d) The comparison between experimental PXRD pattern of Te and simulated PXRD pattern of TeO2 to show the existence of minor impurity of TeO2 in the Te samples.

All of the samples show good crystallinity and experimental patterns match with calculated PXRD patterns. Very low contents of TeO_2 impurity exist in Te samples.
